# IDENTIFICATION OF ADAMTS7 AND CEACAM19 AS CANDIDATE HEALTHY AGING ASSOCIATED GENES

**DOI:** 10.1093/geroni/igz038.382

**Published:** 2019-11-08

**Authors:** Daniel S Evans, Steven R Cummings

**Affiliations:** California Pacific Medical Center Research Institute, San Francisco, California, United States

## Abstract

The goals of the Longevity Genomics research group (www.longevitygenomics.org) are to develop translational strategies to promote healthy human aging based on findings from genomic studies of aging. Our investigation began with the previously reported and publicly available GWAS of the top 10% of parental lifespan in the UK Biobank, reported by Pilling et al. From five loci reported by Pilling et al., 61 candidate longevity-associated genes (LAGs) were identified using FUMA software. We then tested the 61 candidate genes for aging-related trait associations with tissue-specific predicted gene expression in cohort studies of elderly participants using PrediXcan. PrediXcan was applied to three longitudinal cohort studies of elderly individuals: Health ABC, MrOS, and SOF with a total N of 9893 participants. Aging outcomes tested for association were in the following categories: survival and lifespan, kidney function, cognitive function, physical performance tests, self-reported health and disability, diabetes-related traits, cardiovascular-related traits, lung function, and markers of inflammation. After correction for multiple testing, two genes were significantly associated with longevity-related outcomes. Predicted expression of CEACAM19 in the aorta was significantly associated with circulating C-reactive protein (CRP) levels. In addition, predicted expression of ADAMTS7 in the spleen was associated with a measure of lung function, the forced expiratory volume in the first second. Biological mechanisms for these associations are proposed, and our project provides funding opportunities for interested scientists to follow-up these results with laboratory studies (www.longevitygenomics.org/funding).

